# Racial Disparities in Diagnosis of Attention-Deficit/Hyperactivity Disorder in a US National Birth Cohort

**DOI:** 10.1001/jamanetworkopen.2021.0321

**Published:** 2021-03-01

**Authors:** Yu Shi, Lindsay R. Hunter Guevara, Hayley J. Dykhoff, Lindsey R. Sangaralingham, Sean Phelan, Michael J. Zaccariello, David O. Warner

**Affiliations:** 1 Department of Anesthesiology and Perioperative Medicine, Mayo Clinic, Rochester, Minnesota; 2 Department of Health Sciences Research, Mayo Clinic, Rochester, Minnesota; 3 OptumLabs, Cambridge, Massachusetts; 4 Department of Psychiatry and Psychology, Mayo Clinic, Rochester, Minnesota

## Abstract

**Importance:**

There are limited data on the racial disparities in the incidence of attention-deficit/hyperactivity disorder (ADHD) diagnosis in children at the national level.

**Objective:**

To explore differences in rates of diagnosis of ADHD and use of treatment among children by race and ethnicity.

**Design, Setting, and Participants:**

This retrospective cohort study assessed insurance claims data of children born in the US between January 1, 2006, and December 31, 2012, who had continuous insurance coverage for at least 4 years. The last date of follow-up included in the cohort was June 30, 2019. Race/ethnicity designations were based on self-report and included non-Hispanic White, Black, Hispanic, and Asian. Data were analyzed between October 2019 and December 2020.

**Exposures:**

Race and ethnicity.

**Main Outcomes and Measures:**

ADHD diagnosis as defined by *International Classification of Diseases* codes (ninth or tenth editions) and treatment within 1 year of diagnosis, including medication and behavior therapy as defined by billing codes. Data on ADHD diagnosis and treatment were adjusted for sex, region, and household income in a multivariate Cox regression model.

**Results:**

Among 238 011 children in the cohort (116 093 [48.8%] girls; 15 183 [6.7%] Asian, 14 792 [6.2%] Black, 23 358 [9.8%] Hispanic, and 173 082 [72.7%] White children), 11 401 (4.8%) were diagnosed with ADHD. The cumulative incidence at age 12 was 13.12% (95% CI, 12.79%-13.46%). In multivariate Cox regression adjusting for sex, region, and household income, the hazard ratio for Asian children was 0.48 (95% CI, 0.43-0.53); Black children, 0.83 (95% CI, 0.77-0.90); and Hispanic children, 0.77 (95% CI, 0.72, 0.82) compared with White children. In the first year after diagnosis, 516 preschool children (19.4%) received behavioral therapy only, 860 (32.4%) had medications only, 505 (19.0%) had both, and 774 (29.2%) had no claims associated with either option. A higher percentage of school-aged children (2904 [65.6%]) were prescribed medications, and fewer had therapy only (639 [14.4%]) or no treatment at all (884 [20.0%]). Compared with other groups, White children were more likely to receive some kind of treatment. Asian children had the highest odds of receiving no treatment (odds ratio compared with White children, 0.54; 95% CI, 0.42-0.70).

**Conclusions and Relevance:**

Racial and ethnic disparities in the diagnosis and treatment of ADHD are evident. Future study is needed to elucidate the mechanism behind these disparities, with special attention to Asian children. Clinicians should provide racially sensitive care in the evaluation and treatment of ADHD.

## Key Points

**Question:**

Are there racial and ethnic disparities in the diagnosis and treatment of attention-deficit/hyperactivity disorder (ADHD) in children?

**Findings:**

This cohort study of 238 011 children examined the association between race/ethnicity and the diagnosis of ADHD. Asian, Black, and Hispanic children were significantly less likely to be diagnosed with ADHD compared with White children. White children were also more likely to receive treatment for ADHD.

**Meaning:**

These findings suggest that racial and ethnic disparities in the diagnosis and treatment of ADHD are evident.

## Teaser

This cohort study uses a US birth cohort of children covered by commercial insurance to examine the association between race/ethnicity and diagnosis of attention-deficit/hyperactivity disorder (ADHD).

## Introduction

Attention-deficit/hyperactivity disorder (ADHD) affects a large number of children and has long-term effects on their health and learning.^1,2,3^ For example, ADHD is associated with poorer quality of life and higher medical costs.^2,4^ Based on data from national surveys, the prevalence of ADHD in the US appears to have increased in the last 2 decades.^5,6^ However, little is known about the incidence of ADHD diagnosis at the national level. Knowledge about the age at initial diagnosis and the number of children being diagnosed at a certain age could support the formulation of public health policy to improve early interventions such as medical treatments and school services.^7,8,9^

Previous studies suggest that racial and ethnic disparities exist in the diagnosis of ADHD. Although most studies have reported differences among prevalence of ADHD between racial and ethnic groups, there are discrepancies in the direction of the inequality. Black children may have a higher or lower rate compared with non-Hispanic White children depending on the study.^10,11,12,13,14,15^ The underlying mechanism of these disparities is unclear, but it may include socioeconomic and cultural factors, variations in the interpretation of children’s behavior, and the application of diagnostic criteria.^11,16,17,18^ Children from different racial groups may also have different rates of psychiatric comorbidities.^19^

Racial and ethnic disparities may also exist in the treatment of ADHD. According to guidelines for treatment of ADHD, any child aged 4 through 18 years who presents with academic or behavioral problems suggesting ADHD should be evaluated by a primary care clinician.^20^ Once a diagnosis of ADHD is made, behavioral therapy should be the first-line treatment for preschool-aged children and continue to be part of the treatment plan, along with medication starting in elementary school-aged children.^20^ However, it is unclear how many children are treated according to this guideline. Previous reports have indicated that non-Hispanic White children were more likely to take medication for ADHD compared with children in other racial and ethnic groups.^21^ It is unknown if such disparities in treatment occur immediately after the initial diagnosis. Further understanding of how treatment patterns for ADHD may differ based on race, at the time of initial diagnosis and in the early stages of treatment, may help all children receive appropriate evidence-based care.

In this study, we constructed a birth cohort of children from a national commercial insurance–based data set to test the hypotheses that Non-Hispanic White children have higher cumulative incidence of ADHD diagnosis and that they are more likely to be treated with medications within the first year after diagnosis compared with children from other racial or ethnic groups. We also explored whether children had different psychiatric comorbidities associated with ADHD based on race and ethnicity. Unlike previous studies that relied on parental reports, we used clinical diagnostic and billing codes as outcome measures.

## Methods

The Mayo Clinic institutional review board exempted this study from review and informed consent requirements because the study used preexisting and deidentified data. We followed the Strengthening the Reporting of Observational Studies in Epidemiology (STROBE) reporting guideline for cohort studies.

### Study Population

We constructed a retrospective birth cohort of children using deidentified administrative claims data with socioeconomic status information from the OptumLabs Data Warehouse, which includes medical and pharmacy claims, and enrollment records for commercial and Medicare Advantage enrollees. The database contains longitudinal health information on enrollees and patients representing a diverse mixture of ages, ethnicities, and geographical regions across the US. The birth cohort in our analysis included children who were born between January 1, 2006, and December 31, 2012, and had continuous commercial insurance coverage for at least 4 years from birth to the last date of follow-up. In the analysis of treatment for ADHD in the first year after the initial diagnosis, only children who had a least 1 year of insurance coverage beyond the date of ADHD diagnosis were included.

### Outcomes

The primary outcome was the diagnosis of ADHD, which is defined by *International Classification of Diseases, Ninth Revision (ICD-9)*^22^ codes of 314.X or *ICD-10*^23^ codes of F90.X. ADHD with predominantly inattentive type was defined as having *ICD-9* code of 314.00 or *ICD-10* code of F90.0. If a child was diagnosed with ADHD before 3 years of age but did not have any subsequent visits related to ADHD, they were classified as not having ADHD. This decision was made based on the American Academy of Pediatrics clinical practice guideline^20^ that there is insufficient evidence to recommend diagnosis and treatment for children younger than 4 years of age.

Secondary outcomes included both medications and behavior therapies for treatment of ADHD in the clinical setting after the initial date of diagnosis. Medications were identified by having a filled prescription approved for the treatment of ADHD. Psychological behavioral therapy was identified by *Current Procedural Terminology* codes, Healthcare Common Procedure Coding System codes, and revenue codes.

Psychiatric comorbidities among the children who were diagnosed with ADHD, including common internalizing and externalizing disorders, were evaluated separately from date of birth to the date of ADHD diagnosis and from the date of diagnosis to the end of follow-up. Comorbidities were identified using *ICD-9* and/or *ICD-10* codes anywhere within the claim. Only the comorbidities with statistically significant difference based on race were reported.

### Explanatory Variables

Race and ethnicity were based on self-report and were combined as 1 variable and classified as non-Hispanic White (ie, White), non-Hispanic Black (Black), Hispanic, Asian, or other/unknown.^24^ Geographic regions were classified as Midwest, Northeast, South, and West according to the US census. Annual household income in US dollars at the time of last follow-up was categorized as less than $40 000, $40 000 to $74 999, $75 000 to $124 999, $125 000 to $199 999, $200 000 or more, and other/unknown.^24^ Patients with missing race/ethnicity or annual household income were classified into the respective other/unknown categories. A sensitivity analysis was performed by identifying patients who had missing variables and then removing them from the cohort entirely. Removal of these patients did not significantly change the results.

### Statistical Analysis

Differences in baseline characteristics according to ADHD status were examined using a χ^2^ test for categorical variables and a *t* test for continuous variables. In the analysis of ADHD incidence, a child contributed to person-time until the first date of ADHD diagnosis, the end of insurance plan coverage, or the end of the follow-up period (ie, June 30, 2019). Cumulative incidence was calculated based on the number of children who were diagnosed with ADHD over the number of children who contributed to person-time. Incidence over time was depicted using Kaplan-Meier curves. Cox proportional hazards regression was performed to calculate the hazard ratio (HR) of ADHD comparing other racial/ethnic groups with the results from White children. Multivariate Cox regression was then performed to adjust for sex, region, and household income.

In the analyses of psychiatric comorbidities and treatment within the first year of ADHD diagnosis, we restricted analysis to children who had at least 1 year of follow-up after their initial diagnosis. A χ^2^ test was performed when making comparisons among racial groups. *P* values were based on *F* tests. A *P* < .01 was considered statistically significant in 2-sided tests. Analyses were performed using SAS Enterprise Guide version 7.13 (SAS Institute Inc) and Stata version 16.1 (StataCorp). Data analysis was conducted from October 2019 to December 2020.

## Results

Among the 238 011 children in the cohort, 116 093 (48.8%) were girls; 15 183 (6.7%) were Asian, 14 792 (6.2%) were Black, 23 358 (9.8%) were Hispanic, and 173 082 (72.7%) were White children (Table 1). In this cohort, 11 401 children (4.8%) were diagnosed with ADHD during the follow-up period with a mean (SD) age of diagnosis of 6.5 (1.9) years. The overall incidence of ADHD was 69 (95% CI, 68-70) per 10 000 person years (eTable 1 in the Supplement). Compared with children who were not diagnosed with ADHD, children with ADHD had more years of coverage in the data set (mean [SD], 8.8 [2.1] years vs 7.0 [2.2] years; *P* < .001) and were more likely to be boys (8192 [71.9%] vs girls, 3209 [28.1%]), White (8980 [78.8%] vs Black children, 682 [6.0%]), and from the Southern census region (5771 [50.6%] vs Northeast region, 1340 [11.8%]). Approximately half of the diagnoses were made by pediatricians. Other clinicians who made the initial diagnosis included psychologists, family practice providers, psychiatrists, and neurologists.

**Table 1.** Demographic Characteristics of Study Cohort

Characteristic	Children, No. (%)			*P* value
	ADHD		Total (N = 238 011)	
	Yes (n = 11 401)	No (n = 226 610)		
Year of birth				
2006	2208 (19.4)	32 071 (14.2)	34 279 (14.4)	<.001
2007	2185 (19.2)	35 543 (15.7)	37 728 (15.9)	
2008	2022 (17.7)	34 757 (15.3)	36 779 (15.5)	
2009	1699 (14.9)	34 487 (15.2)	36 186 (15.2)	
2010	1415 (12.4)	30 141 (13.3)	31 556 (13.3)	
2011	1104 (9.7)	29 564 (13.0)	30 668 (12.9)	
2012	768 (6.7)	30 047 (13.3)	30 815 (12.9)	
Years of insurance coverage				
Mean (SD)	8.8 (2.1)	7.0 (2.2)	7.1 (2.2)	<.001
Median (IQR)	8.7 (7.2-10.5)	6.6 (5.1-8.3)	6.6 (5.2-8.4)	
Range	4.0-13.0	4.0-13.0	4.0-13.0	
Years from birth to censor date				
Mean (SD)	6.5 (1.9)	7.0 (2.2)	6.9 (2.2)	<.001
Median (IQR)	6.5 (5.3-7.7)	6.6 (5.1-8.3)	6.5 (5.2-8.3)	
Range	0.0-12.9	4.0-13.0	0.0-13.0	
Girls	3209 (28.1)	112 884 (49.8)	116 093 (48.8)	<.001
Race/ethnicity				
White	8980 (78.8)	164 102 (72.4)	173 082 (72.7)	
Asian	365 (3.2)	15 466 (6.8)	15 831 (6.7)	<.001
Black	682 (6.0)	14 110 (6.2)	14 792 (6.2)	
Hispanic	1034 (9.1)	22 324 (9.9)	23 358 (9.8)	
Other/unknown	340 (3.0)	10 608 (4.7)	10 948 (4.6)	
Census region				
Midwest	2800 (24.6)	60 980 (26.9)	63 780 (26.8)	<.001
Northeast	1340 (11.8)	26 088 (11.5)	27 428 (11.5)	
South	5771 (50.6)	97 950 (43.2)	103 721 (43.6)	
West	1490 (13.1)	41 592 (18.4)	43 082 (18.1)	
Household income, $				
<40 000	724 (6.4)	11 066 (4.9)	11 790 (5.0)	<.001
40 000-74 999	1865 (16.4)	32 317 (14.3)	34 182 (14.4)	
75 000-124 999	3118 (27.3)	53 232 (23.5)	56 350 (23.7)	
125 000-199 999	2498 (21.9)	43 529 (19.2)	46 027 (19.3)	
≥200 000	2184 (19.2)	38 103 (16.8)	40 287 (16.9)	
Other/unknown	1012 (8.9)	48 363 (21.3)	49 375 (20.7)	

Abbreviations: ADHD, attention-deficit/hyperactivity disorder; IQR, interquartile range.

At age 4 years, 0.39% (95% CI, 0.36%-0.41%) of the children in the cohort had the diagnosis of ADHD. The number of new diagnoses increased as the children reached school age. The cumulative incidences at ages 6, 8, and 10 years were 2.35% (95% CI, 2.28%-2.42%), 6.62% (95% CI, 6.48%-6.76%), and 10.57% (95% CI, 10.35%-10.80%), respectively. By the age of 12 years, 13.12% (95% CI, 12.79-13.46%) of children who remained in the cohort were diagnosed with ADHD. The incidence for girls was always lower than that for boys at any given age (eFigure in the Supplement).

Cumulative incidence of ADHD by race/ethnicity was highest for White children (eg, age 12 years: 14.19%; 95% CI, 13.79%-14.60% vs Black children, 11.76%; 95% CI, 10.63%-13.01%) and lowest for Asian children (6.08%; 95% CI, 5.25%-7.03%). The Figure displays the adjusted cumulative incidence of ADHD by race and ethnicity after controlling for sex, household income, and region. White children had the highest incidence among the groups. The curves for Black and Hispanic children were similar in shape and slightly lower than that for White children. Asian children had a much lower incidence at all time points.

**Figure.**
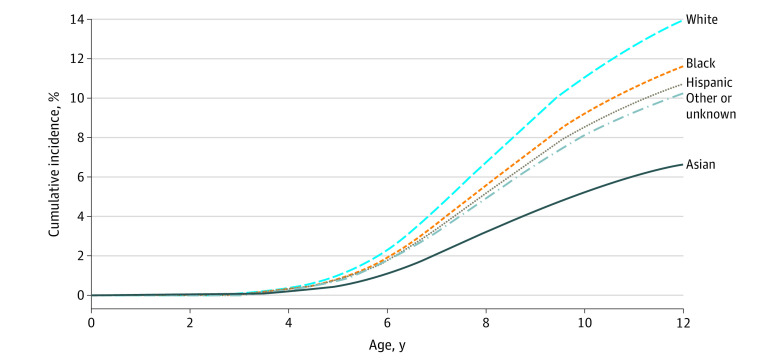
Adjusted Cumulative Incidence by Race and Ethnicity This graph was produced using stcurve following stcox in Stata version 16.1 (StataCorp).

In simple Cox regression, the HR of developing ADHD was 0.47 (95% CI, 0.42-0.52) when comparing Asian with White children (Table 2). In the multivariate Cox regression, the HR for Asian children remained below 0.5 (HR, 0.48; 95% CI, 0.43-0.53), while the HRs for Black and Hispanic children were approximately 0.8 after adjusting for sex, region, and household income (Black children: HR, 0.83; 95% CI, 0.77-0.90; Hispanic children: HR, 0.77; 95% CI, 0.72-0.82). Girls were less likely to be diagnosed compared with boys (HR, 0.40; 95% CI, 0.38-0.41). A lower incidence of ADHD was associated with residing in the Midwest (HR, 0.77; 95% CI, 0.72-0.82) and West (HR, 0.64; 95% CI, 0.60-0.69) and with higher household incomes (eg, <$40 000 vs >$200 000: HR, 1.57; 95% CI, 1.44-1.71).

**Table 2.** Hazard Ratios From Univariate and Multivariate Cox Regression to Predict Incident ADHD

Characteristic	Hazard ratio (95% CI)	
	Univariate model	Multivariate model^a^
Race/ethnicity		
White	1 [Reference]	1 [Reference]
Asian	0.47 (0.42-0.52)	0.48 (0.43-0.53)
Black	0.94 (0.87-1.02)	0.83 (0.77-0.90)
Hispanic	0.84 (0.79-0.90)	0.77 (0.72-0.82)
Other/unknown	0.73 (0.66-0.81)	0.73 (0.66-0.82)
Female sex	0.40 (0.38-0.41)	0.40 (0.38-0.41)
Census region		
Northeast	1 [Reference]	1 [Reference]
Midwest	0.86 (0.81-0.92)	0.77 (0.72-0.82)
South	1.13 (1.06-1.20)	1.06 (1.00-1.13)
West	0.66 (0.62-0.71)	0.64 (0.60-0.69)
Household income, $		
≥200 000	1 [Reference]	1 [Reference]
125 000-199 999	1.14 (1.08-1.21)	1.16 (1.09-1.23)
75 000-124 999	1.31 (1.24-1.39)	1.33 (1.26-1.41)
40 000-74 999	1.40 (1.32-1.49)	1.44 (1.35-1.54)
<40 000	1.52 (1.39-1.65)	1.57 (1.44-1.71)
Other/unknown	1.22 (1.13-1.32)	1.29 (1.19-1.39)

Abbreviation: ADHD, attention-deficit/hyperactivity disorder.

^a^ The multivariate model included adjustments for sex, region, and household income.

Children were often diagnosed with other disorders prior to their ADHD diagnosis (Table 3), with the frequencies of diagnoses varying by race and ethnicity. Asian children were more likely to be diagnosed with speech sound disorders (97 [26.6%] vs White children, 1427 [15.9%]; *P* < .001), unspecified neurodevelopmental disorders (54 [14.8%] vs White children, 784 [8.7%]; *P* < .001), and autism spectrum disorder (62 [17.0%] vs White children, 527 [5.9%]; *P* < .001) compared with other groups. White children were more likely to be diagnosed with unspecified anxiety disorders (500 [5.6%] vs Black children, 29 [4.3%]; *P* = .01) and adjustment disorder (1085 [12.1%] vs Black children, 68 [10.0%]; *P* = .001). After a diagnosis of ADHD was made, White children were more likely to be subsequently diagnosed with generalized anxiety disorders (815 children [9.1%]) and unspecified anxiety disorders (1312 children [14.6%]). Asian children continued to be more likely to receive a diagnosis of autism spectrum disorder (85 children [23.3%]). There were no significant differences based on race in the frequency of other diagnoses, such as depressive disorders, disruptive disorders, or oppositional defiant disorder.

**Table 3.** Psychiatric Comorbidities Before and After Diagnosis of ADHD^a^

Comorbidity	Children, No. (%)						*P* value^b^
	White (n = 8980)	Asian (n = 365)	Black (n = 682)	Hispanic (n = 1034)	Other/unknown (n = 340)	Total (N = 11 401)	
**Pre-ADHD diagnosis**							
Speech sound disorder	1427 (15.9)	97 (26.6)	104 (15.2)	196 (19.0)	54 (15.9)	1878 (16.5)	<.001
Unspecified anxiety disorder	500 (5.6)	18 (4.9)	29 (4.3)	32 (3.1)	14 (4.1)	593 (5.2)	.01
Unspecified neurodevelopmental disorder	784 (8.7)	54 (14.8)	50 (7.3)	105 (10.2)	37 (10.9)	1030 (9.0)	<.001
Autism spectrum disorder	527 (5.9)	62 (17.0)	43 (6.3)	68 (6.6)	22 (6.5)	722 (6.3)	<.001
Adjustment disorder	1085 (12.1)	31 (8.5)	68 (10.0)	94 (9.1)	27 (7.9)	1305 (11.4)	.001
**Post-ADHD diagnosis**							
Generalized anxiety disorder	815 (9.1)	19 (5.2)	39 (5.7)	57 (5.5)	24 (7.1)	954 (8.4)	<.001
Unspecified anxiety disorder	1312 (14.6)	46 (12.6)	64 (9.4)	117 (11.3)	42 (12.4)	1581 (13.9)	<.001
Autism spectrum disorder	912 (10.2)	85 (23.3)	80 (11.7)	139 (13.4)	44 (12.9)	1260 (11.1)	<.001

Abbreviation: ADHD, attention-deficit/hyperactivity disorder.

^a^ Comorbidities evaluated in the study include common pediatric internalizing and externalizing disorders.

^b^ *P* values are based on *F* tests.

In the first year after ADHD diagnosis, 516 of 2655 preschool children (19.4%) received behavioral therapy only, 860 (32.4%) had medications only, 505 (19.0%) had both, and 774 (29.2%) had no claims associated with either option (Table 4). When the diagnosis was made during school age, a higher percentage of children were prescribed medications (2904 [65.6%]), and fewer had therapy only (639 [14.4%]) or no treatment at all (884 [20.0%]). Compared with other groups, White children were more likely to receive some kind of treatment. Compared with White children, Asian children had the highest odds of receiving no treatment (odds ratio, 0.54; 95% CI, 0.42-0.70) (eTable 2 in the Supplement).

**Table 4.** Treatments Within First Year After ADHD Diagnosis

Treatment	Children, No. (%)									*P* value^a^
	White	Asian	Black			Hispanic		Other/unknown	Total	
**ADHD diagnosis age <6 y**										
Total^b^	2032 (76.5)	107 (4.0)		178 (6.7)	250 (9.4)		88 (3.3)		2655 (100)	
Both medication and therapy	404 (19.9)	11 (10.3)		36 (20.2)	36 (14.4)		18 (20.5)		505 (19.0)	<.001
Medication only	702 (34.5)	16 (15.0)		60 (33.7)	57 (22.8)		25 (28.4)		860 (32.4)	
Therapy only	390 (19.2)	31 (29.0)		23 (12.9)	56 (22.4)		16 (18.2)		516 (19.4)	
No treatment	536 (26.4)	49 (45.8)		59 (33.1)	101 (40.4)		29 (33.0)		774 (29.2)	
**ADHD diagnosis age ≥6 y**										
Total^b^	3577 (80.8)	111 (2.5)		240 (5.4)	383 (8.7)		116 (2.6)		4427 (100)	
Both medication and therapy	819 (22.9)	18 (16.2)		55 (22.9)	51 (13.3)		25 (21.6)		968 (21.9)	<.001
Medication only	1592 (44.5)	39 (35.1)		101 (42.1)	164 (42.8)		40 (34.5)		1936 (43.7)	
Therapy only	516 (14.4)	17 (15.3)		33 (13.8)	54 (14.1)		19 (16.4)		639 (14.4)	
No treatment	650 (18.2)	39 (35.1)		51 (21.3)	114 (29.8)		32 (27.6)		884 (20.0)	

Abbreviation: ADHD, attention-deficit/hyperactivity disorder.

^a^ *P* values are based on *F* tests.

^b^ Percentages in this row are out of the total number of children in the cohort.

## Discussion

The major contributions of this study to the current literature include providing findings that suggest (1) significant racial disparities in ADHD diagnosis in privately insured children, including Asian children; (2) racial disparities in treatment, including evidence for gaps between guidelines and clinical practice; and (3) differing patterns of comorbidities accompanying ADHD diagnosis according to race.

A limited number of previous studies have reported the incidence of diagnosis of ADHD.^25,26,27^ In our study, the cumulative incidence increased steadily as children entered school, with 10% of children diagnosed before age 10 years. Our study is consistent with a 2004 US population-based study by Barbaresi et al.^25^ Using a cohort in Olmsted County, Minnesota, their cumulative incidence at age 13 years was approximately 10%, which is comparable with our estimate of 13% at age 12 years. Our findings are also consistent with previous studies that reported boys are more than twice as likely to be diagnosed with ADHD than girls and that there are higher rates of ADHD in children from families with lower income.^13,14^ Regional differences in diagnosis rates were also similar to previous studies, which also found higher rates of ADHD diagnosis in the South and Midwest regions of the US.^6,15^

Racial disparities in prevalence of ADHD diagnosed based on parental reports have been reported in several national surveys. In both the National Health Interview Survey^14^ and National Survey of Children’s Heath,^15^ Black children had higher prevalence compared with White children, whereas Hispanic children were 35% less likely to be diagnosed compared with White children. According to the Early Childhood Longitudinal Study, the incidence of diagnosis of ADHD for Hispanic children was also significantly lower than that for non-Hispanic children.^27^ However, in contrast to the National Health Interview Survey and National Survey of Children’s Health, the HR comparing ADHD diagnosis for Black with White children was 0.3. The racial disparities in these national surveys persisted after controlling for socioeconomic factors. When ADHD diagnosis was made according to *Diagnostic and Statistical Manual of Mental Disorders* (Fifth Edition)^28^ using information obtained from parental interview, a study with the National Health and Nutrition Examination Survey also showed higher prevalence of ADHD in White children (12.65%) aged 12 to 15 years compared with Hispanic (7.11%) or Black (7.69%) children.^29^ In comparison, we found a smaller but consistent difference between White and both Black and Hispanic children, with the latter being less likely to be diagnosed. In our study, all children had coverage by commercial insurance, which decreased the potential effect of access to health care on ADHD diagnosis. Fewer studies of ADHD have examined Asian children as a separate racial group. Our finding that this group had the lowest incidence of ADHD is consistent with 2 studies of children covered by the Kaiser Permanente health plan in California.^12,13^

The cause of the disparities in ADHD diagnosis according to race and ethnicity is not fully understood. Eiraldi et al^30^ proposed a model on the care-seeking pathway of children with ADHD and provided a conceptual framework for understanding racial differences in ADHD diagnosis and treatment. This model included multiple factors that may influence problem recognition, making the decision to seek help, service selection, and service utilization. For example, Coker et al^31^ suggested that Black children had more symptoms consistent with ADHD based on questionnaires but were less likely to have been clinically diagnosed. Because of the variation in symptoms of ADHD, cultural values may impact the perceptions of such behaviors.^32,33^ Our findings provide some evidence that Asian parents brought their children for clinical evaluation for reasons that differed compared with other racial groups, as reflected by the differences in comorbidities preceding or subsequent to ADHD diagnosis among racial and ethnic groups: Asian children were found to have higher rates of speech language disorder and autism spectrum disorder while White children had more anxiety and adjustment disorders. It is also likely that patients’ concerns about racism play some role in influencing their willingness to approach the health care system.^34^

The disparities in treatment among children who were diagnosed with ADHD may also reflect the results of parental care-seeking preferences. Asian and Hispanic children were less likely to receive medication treatment than White and Black children. However, the percentage of Asian children receiving psychotherapy was not significantly lower than other groups, which is different than a 2013 study^13^ finding that Asian children with ADHD were less likely to use mental health services. Disparities in mental health service in children continue to be a public health issue.^35^ Health care professionals may also contribute to the racial disparities in diagnosis and treatment. Stereotype and bias, both explicit and implicit, have been increasingly recognized as factors potentially contributing to physicians’ clinical decision-making.^36,37,38^ It is possible, for example, that identical behavior displayed by Black and non-Hispanic White children may be interpreted differently based on race-based expectations for the behavior of children, and thus, behavior that is identified as disordered in White children might be inappropriately interpreted as normal in Black children.

In our cohort, more than half of children who were diagnosed during preschool years were taking ADHD medications. Although it is unknown whether those children had received failed behavioral therapy treatment before the initiation of medications, this finding may suggest deviation from the American Academy of Pediatrics clinical practice guidelines. Most school-aged children were treated with medications, which is consistent with previous reports.^15,21^

### Limitations

This study has several limitations. First, we used *ICD* codes to identify ADHD cases. Because administrative data were collected for billing purposes rather than research, the presence of *ICD* codes may not always indicate true clinical diagnosis. The utility of *ICD* codes in ascertaining ADHD has been evaluated in previous studies,^39,40,41,42^ where it was shown to have a high sensitivity, specificity, and positive predictive value.^40,42^ However, we still might have mistakenly assigned ADHD status to a small number of children. Second, our birth cohort of children was from a national commercial insurance database and may not be representative of all children in the US. However, a 2018 report^21^ on children aged 2 to 17 years who were covered by Medicaid in New York state suggested 5.4% were diagnosed with ADHD, which is similar to our finding of 4.8%. Third, we had no data on child-level information, such as behavioral symptoms, and were unable to comment on the possibilities of under- or overdiagnosis of ADHD or any of the psychiatric comorbidities. Fourth, in the evaluation of psychotherapy for ADHD, we had no data on any therapies that were not covered by the insurance plan and thus did not result in a claim in the data set. As a result, the number of children in therapy is likely underestimated. We are also unable to comment on any services that children received from their schools. Fifth, we were only able to control for a limited number of variables in our multivariate analysis and could not rule out the effect of unmeasured confounders.

## Conclusions

In a national birth cohort of children covered by commercial insurance, racial and ethnic disparities in the diagnosis and treatment of ADHD were evident. Future study is needed to elucidate the mechanism behind these disparities. Clinicians should provide racially and culturally sensitive care in the evaluation and treatment of ADHD to ensure all children receive appropriate care.

## Notes

Supplement. **eTable 1.** Incidence of ADHD Diagnosis Based on Patient Characteristics **eTable 2.** Odds Ratios of Receiving ADHD Treatment by Race/Ethnicity, Adjusting for Sex, Census Region, and Annual Household Income **eFigure.** Cumulative Risk of ADHD Diagnosis by Gender
