# Supplementary material for: Racial Disparities in Diagnosis of Attention-Deficit/Hyperactivity Disorder in a US National Birth Cohort
Source: JAMA Netw Open. 2021 Mar 1;4(3):e210321. doi: 10.1001/jamanetworkopen.2021.0321 (PMC7921900; doi:10.1001/jamanetworkopen.2021.0321)
Supplement: Supplement. — eTable 1. Incidence of ADHD Diagnosis Based on Patient Characteristics eTable 2. Odds Ratios of Receiving ADHD Treatment by Race/Ethnicity, Adjusting for Sex, Census Region, and Annual Household Income eFigure. Cumulative Risk of ADHD Diagnosis by Gender [file jamanetwopen-e210321-s001.pdf]

Supplemental Online Content

Shi Y, Hunter Guevara LR, Dykhoff HJ, et al. Racial disparities in diagnosis of attention-deficit/hyperactivity disorder in a US national birth cohort. *JAMA Netw Open*. 2021;4(3):e210321. doi:10.1001/jamanetworkopen.2021.0321

eTable 1. Incidence of ADHD Diagnosis Based on Patient Characteristics

eTable 2. Odds Ratios of Receiving ADHD Treatment by Race/Ethnicity, Adjusting for Sex, Census Region, and Annual Household Income

eFigure. Cumulative Risk of ADHD Diagnosis by Gender

This supplemental material has been provided by the authors to give readers additional information about their work.

© 2021 Shi Y et al. *JAMA Network Open.*

eTable 1. Incidence of ADHD diagnosis based on patient characteristics

	Total number of patients in group	Number of ADHD cases	Number of person-years (sum of follow-up time in years)	Incidence rate per 1000 person-years
Sex				
Female	116,093	3,209	811,753.8	3.953
Male	121,918	8,192	840,436.6	9.747
Race/ethnicity				
Asian	15,831	365	108,690.6	3.358
Black	14,792	682	101,085.8	6.747
Hispanic	23,358	1,034	163,577.7	6.321
Other/unknown	10,948	340	71,094.0	4.782
White	173,082	8,980	1,207,742.3	7.435
Census region				
Midwest	63,780	2,800	445,453.6	6.286
Northeast	27,428	1,340	188,347.2	7.115
South	103,721	5,771	714,985.0	8.071
West	43,082	1,490	303,404.6	4.911
Household income				
<$40K	11,790	724	82,850.9	8.739
$40K-$74,999	34,182	1,865	236,713.2	7.879
$75K-$124,999	56,350	3,118	402,531.5	7.746
$125K-$199,999	46,027	2,498	343,738.8	7.267
$200K+	40,287	2,184	318,434.3	6.859
Other/unknown	49,375	1,012	267,921.7	3.777

© 2021 Shi Y et al. *JAMA Network Open.*

eTable 2. Odds ratio (OR) of receiving ADHD treatment by race/ethnicity, adjusting for sex, census region, and annual household income

	OR (95% CI)
**Race**	
White	Ref
Asian	0.54 (0.42, 0.70)
Black	0.79 (0.64, 0.98)
Hispanic	0.57 (0.48, 0.67)
Other/Unknown	0.69 (0.52, 0.91)
**Sex**	
Female	0.91 (0.82, 1.02)
**Census Region**	
Northeast	Ref
Midwest	2.95 (2.48, 3.51)
South	2.12 (1.82, 2.45)
West	1.63 (1.35, 1.96)
**Household Income**	
>$ 200K	Ref
$125K-$199,999	0.93 (0.79, 1.09)
$75K-$124,999	0.88 (0.75, 1.02)
$40K-$74,999	0.75 (0.63, 0.89)
<$40K	0.90 (0.71, 1.14)
Other/Unknown	0.56 (0.45, 0.70)

© 2021 Shi Y et al. *JAMA Network Open.*

eFigure

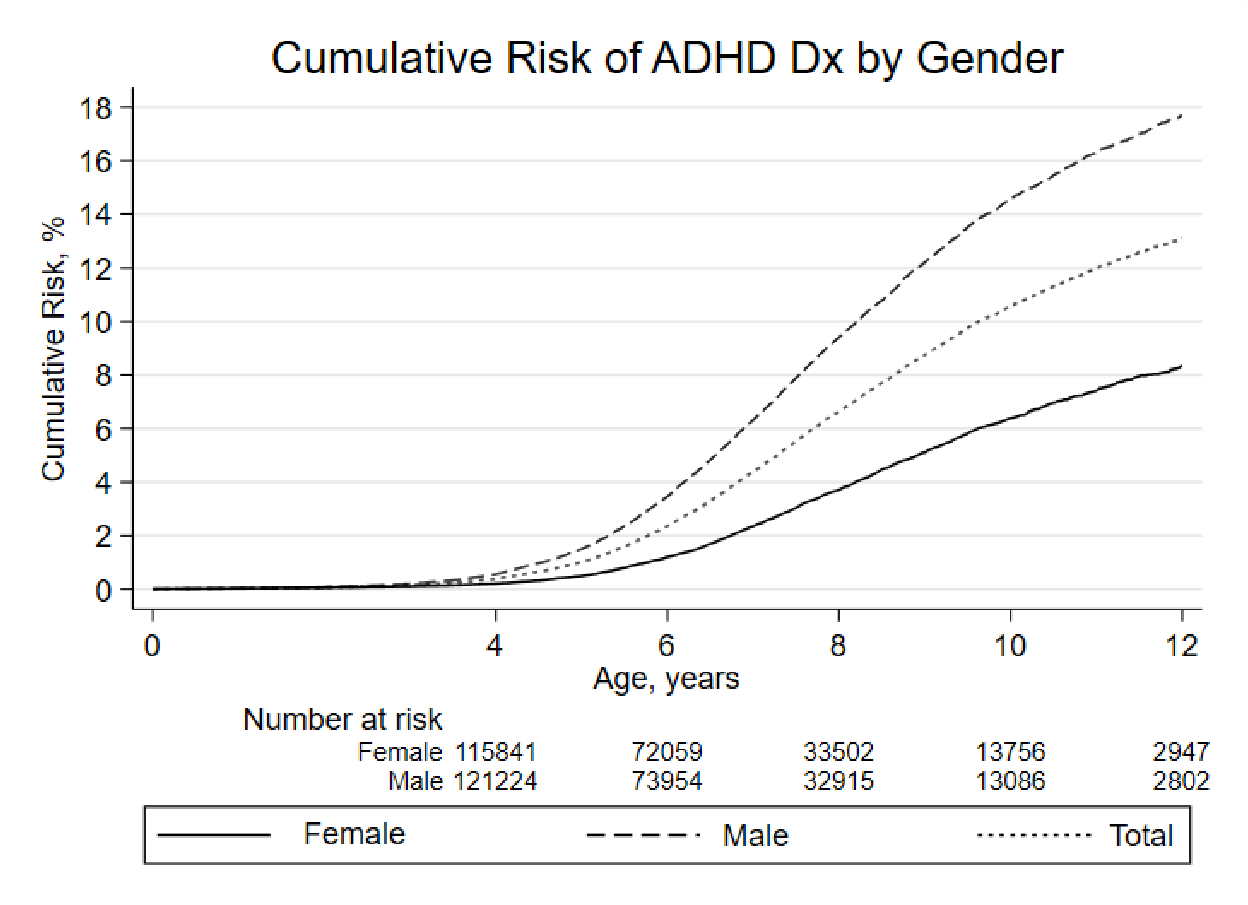

© 2021 Shi Y et al. *JAMA Network Open.*
